# Phase-transforming mechanical metamaterials with dynamically controllable shape-locking performance

**DOI:** 10.1093/nsr/nwad192

**Published:** 2023-07-08

**Authors:** Yiding Zhong, Wei Tang, Huxiu Xu, Kecheng Qin, Dong Yan, Xujun Fan, Yang Qu, Zhaoyang Li, Zhongdong Jiao, Huayong Yang, Jun Zou

**Affiliations:** State Key Laboratory of Fluid Power and Mechatronic Systems, Zhejiang University, Hangzhou 310027, China; School of Mechanical Engineering, Zhejiang University, Hangzhou 310027, China; State Key Laboratory of Fluid Power and Mechatronic Systems, Zhejiang University, Hangzhou 310027, China; School of Mechanical Engineering, Zhejiang University, Hangzhou 310027, China; Institute of Process Equipment, College of Energy Engineering, Zhejiang University, Hangzhou 310027, China; State Key Laboratory of Fluid Power and Mechatronic Systems, Zhejiang University, Hangzhou 310027, China; School of Mechanical Engineering, Zhejiang University, Hangzhou 310027, China; State Key Laboratory of Fluid Power and Mechatronic Systems, Zhejiang University, Hangzhou 310027, China; School of Mechanical Engineering, Zhejiang University, Hangzhou 310027, China; State Key Laboratory of Fluid Power and Mechatronic Systems, Zhejiang University, Hangzhou 310027, China; School of Mechanical Engineering, Zhejiang University, Hangzhou 310027, China; State Key Laboratory of Fluid Power and Mechatronic Systems, Zhejiang University, Hangzhou 310027, China; School of Mechanical Engineering, Zhejiang University, Hangzhou 310027, China; State Key Laboratory of Fluid Power and Mechatronic Systems, Zhejiang University, Hangzhou 310027, China; School of Mechanical Engineering, Zhejiang University, Hangzhou 310027, China; State Key Laboratory of Fluid Power and Mechatronic Systems, Zhejiang University, Hangzhou 310027, China; School of Mechanical Engineering, Zhejiang University, Hangzhou 310027, China; State Key Laboratory of Fluid Power and Mechatronic Systems, Zhejiang University, Hangzhou 310027, China; School of Mechanical Engineering, Zhejiang University, Hangzhou 310027, China; State Key Laboratory of Fluid Power and Mechatronic Systems, Zhejiang University, Hangzhou 310027, China; School of Mechanical Engineering, Zhejiang University, Hangzhou 310027, China; State Key Laboratory of Fluid Power and Mechatronic Systems, Zhejiang University, Hangzhou 310027, China; School of Mechanical Engineering, Zhejiang University, Hangzhou 310027, China

**Keywords:** active mechanical metamaterials, shape locking, energy storage, liquid–vapor phase transform, soft robotics

## Abstract

Active mechanical metamaterials with customizable structures and deformations, active reversible deformation, dynamically controllable shape-locking performance and stretchability are highly suitable for applications in soft robotics and flexible electronics, yet it is challenging to integrate them due to their mutual conflicts. Here, we introduce a class of phase-transforming mechanical metamaterials (PMMs) that integrate the above properties. Periodically arranging basic actuating units according to the designed pattern configuration and positional relationship, PMMs can customize complex and diverse structures and deformations. Liquid–vapor phase transformation provides active reversible large deformation while a silicone matrix offers stretchability. The contained carbonyl iron powder endows PMMs with dynamically controllable shape-locking performance, thereby achieving magnetically assisted shape locking and energy storing in different working modes. We build a theoretical model and finite element simulation to guide the design process of PMMs, so as to develop a variety of PMMs with different functions suitable for different applications, such as a programmed PMM, reconfigurable antenna, soft lens, soft mechanical memory, biomimetic hand, biomimetic flytrap and self-contained soft gripper. PMMs are applicable to achieve various 2D deformations and 2D-to-3D deformations, and integrate multiple properties, including customizable structures and deformations, active reversible deformation, rapid reversible shape locking, adjustable energy storing and stretchability, which could open a new application avenue in soft robotics and flexible electronics.

## Teaser

Soft phase-transforming mechanical metamaterials with customizable structures and deformations, active reversible deformation, rapid reversible shape locking, adjustable energy storing and stretchability are highly suitable for applications in soft robotics and flexible electronics.

## INTRODUCTION

Mechanical metamaterials composed of periodically arranged tailored structural units show excellent properties beyond natural materials [1–3] but these properties often cannot be adjusted after fabrication, which limits the expansion of their applications. In recent years, by combining mechanical metamaterials with flexible smart materials, active mechanical metamaterials [4] that can change their shape, properties and functions under external stimuli have attracted tremendous research interest. A variety of smart soft materials with different actuation technologies, including shape memory polymers [5–7], hydrogels [8,9], soft pneumatic actuators [10,11], magnetically actuated soft materials [12–14], etc., have been reported for the design of active mechanical metamaterials. Among them, active mechanical metamaterials using shape memory polymers can lock shape, but lack stretchability and active reversible deformation capabilities. Although active mechanical metamaterials using hydrogels possess good biocompatibility, their applications are mainly limited to aqueous media. Pneumatic active mechanical metamaterials are simple in design and easy to implement but require external bulky air pumps/compressors. Magnetically actuated active mechanical metamaterials have a fast response speed but require complex external devices to provide the actuating magnetic field with adjustable direction and strength, and the magnetic particles in them often need to be magnetized to orient the magnetic domains, which increases the complexity of fabrication. Magnetically actuated active mechanical metamaterials using magnetic shape memory polymers [14,15] can also achieve shape locking, but the locking or unlocking process is relatively slow and lacks stretchability. Among various smart soft materials, liquid–vapor phase transition composites [16–20] obtained by mixing low-boiling-point fluid (LBPF) into silicone have both the high stretchability of the silicone matrix and the reversible large volume change of the liquid–vapor phase transformation, eliminating the need for external air sources or aqueous medium, and their fabrication methods are simple and convenient. Hence, if liquid–vapor phase transition composites are used to develop active mechanical metamaterials, it is expected to further expand the properties, functions and applications of active mechanical metamaterials. However, the structural forms and deformation types of the existing liquid–vapor phase transition composites are still relatively simple and limited due to the limitations in their steric configurations. Liquid–vapor phase transition composites with complex and diverse structural forms and deformation types realized by periodically combining basic actuating units with compatible configurations remain to be explored to address this challenge. Also, it is a key challenge to design various active mechanical metamaterials by using liquid–vapor phase transition composites.

Furthermore, active mechanical metamaterials that combine active reversible deformation, rapid reversible shape locking, adjustable energy storage and stretchability are highly suitable for numerous applications such as soft robotics [21,22], flexible electronics [23,24] and mechanical logic [25,26]. However, there is currently a lack of reports on integrating these properties in a single active mechanical metamaterial system due to the mutual conflict of these properties. Liquid–vapor phase transition composites exhibit active reversible deformation and stretchability but are restricted by the need for continuous energy consumption to maintain the actuated shape (i.e. lack of shape locking) and the relatively slow response speed. Existing techniques for the shape locking of active mechanical metamaterials mainly utilize the stiffness changes of materials at different temperatures, such as using shape memory polymers [5,7] or low-melting-point alloys [27–29], but these techniques affect the stretchability, exhibit slow locking or unlocking speeds and often require external forces to achieve deformation and shape programming, lacking active reversible deformation. Inspired by some natural organisms such as flytraps [30] and salamanders [31], fast motion can be achieved through elastic energy storage and release, so if the energy-storage performance can be integrated into active mechanical metamaterials based on liquid–vapor phase transition composites, their rapid response can be achieved. Although there are some flexible mechanical metamaterials using energy-storage methods based on bistable or multistable structures in the existing literature [25,26,32], on the one hand, these structures can only switch between a limited few preset states, which affects the functional scalability; on the other hand, these studies are often not active mechanical metamaterials with active reversible deformation.

Here, we design a class of phase-transforming mechanical metamaterials (PMMs) with customizable structures and deformations, integrating active reversible deformation, dynamically controllable shape-locking performance and stretchability. The dynamically controllable shape-locking performance of PMMs endows them with rapid reversible shape locking and adjustable energy storing. The proposed PMMs consist of a series of bilayer basic actuating units arranged periodically in different ways, which include an active layer using magnetically responsive liquid–vapor phase transition composites and a magnetically responsive strain-limiting layer. By controlling the pattern configuration of the PMMs and the positional relationship between the PMMs and their basic actuating units, PMMs can customize complex and diverse structural forms and deformation types, including a variety of 2D deformations and 2D-to-3D deformations. Theoretical analysis and finite element simulation are used to guide the design process of PMMs. Benefitting from the reversible liquid–vapor phase transform of LBPF and the elasticity of the silicone matrix, PMMs can achieve reversible active deformation and recovery during heating and cooling. The added carbonyl iron powder (CIP) endows the PMMs with the ability to lock deformation or store elastic potential energy in the magnetic field provided by the magnets under different working modes. The rapid reversible shape-locking properties of PMMs eliminate continuous energy consumption without compromising stretchability, while their adjustable energy-storage and energy-release properties enable rapid and fully adjustable deformation. Based on the above properties of PMMs, we demonstrate a wide range of function enhancements and applications, including a programmed PMM with a local prestretch design, reconfigurable antenna with on-demand deformation and shape locking, soft lens with tunable imaging, soft mechanical memory, biomimetic hand with programmable deformation and gesture locking, biomimetic flytrap with rapid response and self-contained soft gripper with rapid response, illustrating their potential for generalization in soft robotics and flexible electronics.

## RESULTS

### Design and working principles

**Figure 1.**
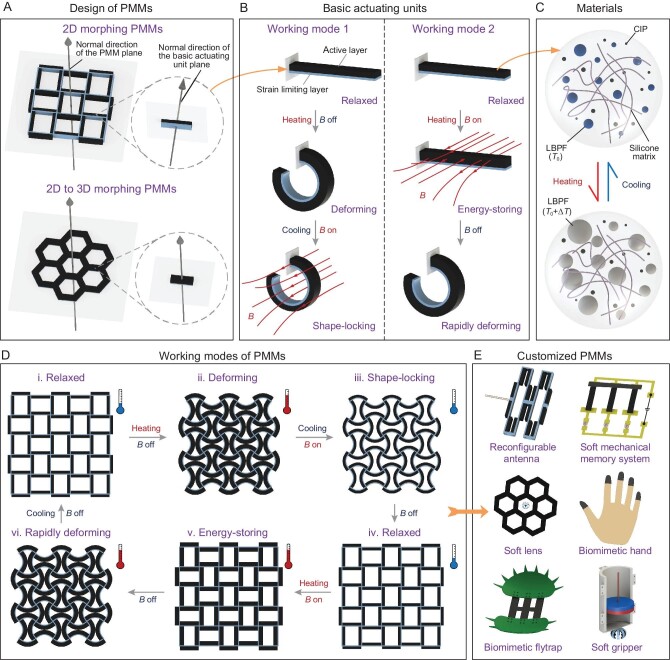
Design and working principle of PMMs. (A) Design principles of 2D and 2D-to-3D morphing PMMs. (B) Working mechanisms of the basic actuating unit of the PMMs, including deformation and shape locking in working Mode 1 and energy storage and rapid deformation in working Mode 2. (C) Deformation principle of magnetically responsive liquid–vapor phase transition composites used in active layers. (D) Schematic sequence showing the PMM cyclically changing between states including relaxed, deforming, shape locking, energy storing and rapid deforming, combining two working modes. (E) Customized PMMs used in flexible electronics and soft robotics.

The PMMs consist of a series of bilayer basic actuating units arranged periodically with customized patterns. As shown in Fig. 1A, by designing the positional relationship between the PMMs and their basic actuating units, we can control the deformation type of the PMMs. In the initial state, when the normal direction of the PMM plane is designed to be perpendicular to the normal direction of its basic actuating unit planes, a 2D morphing PMM can be obtained. When the normal direction of the PMM plane is designed to be parallel to the normal direction of its basic actuating unit planes, a 2D-to-3D morphing PMM can be obtained. The bilayer basic actuating unit includes an active layer using magnetically responsive liquid–vapor phase transition composites and a magnetically responsive strain-limiting layer (Fig. 1B). The magnetically responsive liquid–vapor phase transition composites constituting the active layer, with the microscopic image shown in Supplementary Fig. S1, were prepared by fully mixing LBPF, CIP and silicone, and then pouring them into molds for curing. As shown in Supplementary Fig. S2 and Supplementary Movie S1, the silicone matrix endowed the PMMs with high stretchability. The PMMs were fabricated by bonding a number of active layers and strain-limiting layers in a designed pattern and the detailed fabrication process of the PMMs is shown in Supplementary Fig. S3. As shown in Fig. 1C, when heated, the LBPF microdroplets contained in the magnetically responsive liquid–vapor phase transition composites vaporize and squeeze the surrounding silicone matrix, causing the overall volume of the composites to expand significantly, while due to the elasticity of the silicone matrix itself, the composites can recover their original state with the liquefaction of the LBPF after cooling. As shown in Fig. 1B, in the working Mode 1, first heating with the magnetic field (*B*) turned off, the volume expansion of the active layer would lead to a deformation mismatch between the active layer and the strain-limiting layer, thereby causing bending deformation of the basic actuating unit. The heating was then stopped and *B* was turned on because, under *B*, the CIP in the composites would be affected by the magnetic force, and even if the magnetically responsive liquid–vapor phase transition composites were cooled, the shape of the basic actuating unit could still be locked in the bending deformation state. Because *B* is provided by magnets, the shape-locking state requires no continuous energy consumption. At this time, if *B* was turned off, the basic actuating unit would return to the initial flat shape. In working Mode 2, first heating with *B* turned on, although the volume of the magnetically responsive liquid–vapor phase transition composites expanded, due to the magnetic force on the CIP in the composites, the shape of the basic actuating unit could still be locked in the initial flat shape. At the same time, during this process, the volume expansion of the active layer was converted into elastic potential energy and stored in the basic actuating unit. When heating was stopped and *B* was turned off, the elastic potential energy stored in the previous stage would be quickly released, resulting in rapid bending deformation of the basic actuating unit. As shown in Fig. 1D, based on the above two working modes, taking the quadrilateral lattice as an example, the PMMs composed of a series of the above-mentioned basic actuating units can cyclically change among states including relaxed, deforming, shape locking, energy storing and rapid deforming. The shape locking of the PMM mainly means that the curvature of its basic actuating units in the shape-locking state is essentially the same as that in the deforming state. Compared with the deforming state when heated, the magnetically responsive liquid–vapor phase transition composites used for the active layers shrink in volume after cooling, so the shapes of the PMMs in the deforming state and the shape-locking state are not exactly the same. The PMMs can perform complex and diverse deformation that is active and reversible according to the customized pattern. Thanks to the above properties and capabilities of PMMs, as shown in Fig. 1E, various customized PMMs can be used to construct a series of applications in flexible electronics and soft robotics.

### Theoretical analysis and simulation

**Figure 2.**
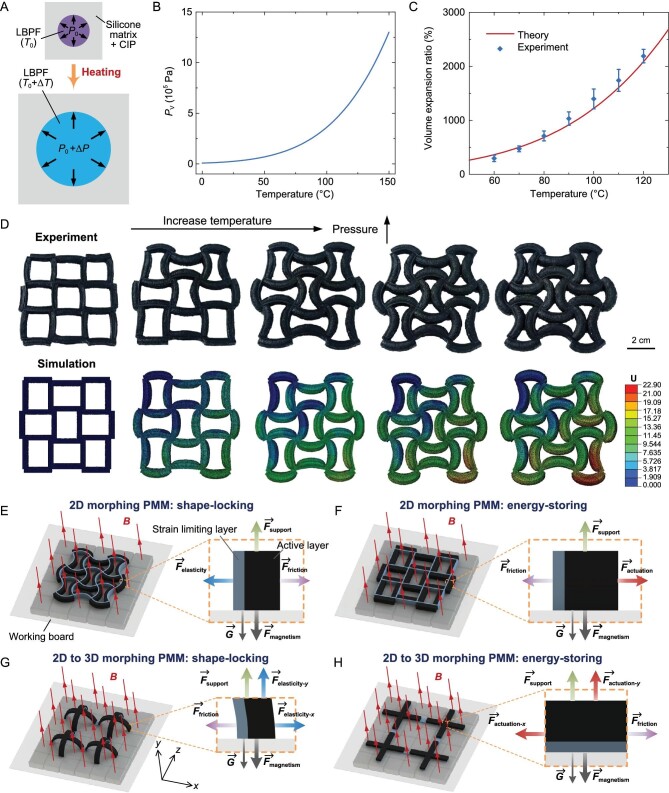
Analysis of deformation and shape locking of PMMs. (A) Schematic illustration of heating-induced expansion of a single LBPF bubble in magnetically responsive liquid–vapor phase transition composites. (B) The saturated vapor pressure property of LBPF composed of equal volumes of ethanol and Novec 7000. (C) Variation in volume expansion ratio of magnetically responsive liquid–vapor phase transition composites with temperature. (D) Finite element simulation and experiment of deformation of the PMM with a quadrilateral lattice. Schematic diagram of the force analysis of (E) the 2D morphing PMM under shape-locking state, (F) the 2D morphing PMM under energy-storing state, (G) the 2D-to-3D morphing PMM under shape-locking state and (H) the 2D-to-3D morphing PMM under energy-storing state.

In order to quantitatively predict the deformation characteristics of magnetically responsive liquid–vapor phase transition composites during heating, we carried out the following theoretical analysis. We regard the composites as a porous material containing many microchambers subjected to internal pressure and we assume that the spherical microchambers containing LBPF are uniformly distributed in the silicone matrix. As shown in Fig. 2A, relative to the reference state (*T*_0_ = 20°C), when the temperature rises Δ*T*, due to the liquid–vapor phase transform of the LBPF contained, the internal pressure change Δ*P* of the microchamber is described by the saturated vapor pressure *P*_v_(*T*) of the LBPF:


(1) \begin{eqnarray*}\Delta P = {P}_{\rm v}\left( {{T}_0 + \Delta T} \right) - {P}_{\rm v}\left( {{T}_0} \right).\end{eqnarray*}

The LBPF used here is a mixture of ethanol and Novec 7000. According to Raoult's law, its saturated vapor pressure is:


(2) \begin{eqnarray*}{P}_{\rm{v}} = \frac{{{n}_{{\rm{ethanol}}}{P}_{{\rm{v}}{-}{{\rm ethanol}}} + {n}_{{\rm{Novec}}}{P}_{{\rm{v{-}Novec}}}}}{{{n}_{{\rm{ethanol}}} + {n}_{{\rm{Novec}}}}},\end{eqnarray*}

where *n*_ethanol_ and *n*_Novec_ are the amount of substance of the two components, and *P*_v-ethanol_ and *P*_v-Novec_ are the saturated vapor pressures of the two components. The calculated result of the saturated vapor pressure *P*_v_ is shown in Fig. 2B. According to the mechanical model of the liquid–vapor phase transition composites in [19], the relative volume change ${\bar{\varepsilon }}_{{\rm{kk}}}$ after heating and expansion is:


(3) \begin{eqnarray*}{\bar{\varepsilon }}_{{\rm{kk}}} = \frac{{V - {V}_0}}{{{V}_0}} = 3\left( {\frac{f}{{1 \!-\! f}}\frac{{3\kappa + 4\mu }}{{12\kappa \mu }}\Delta P + \alpha \Delta T} \right),\end{eqnarray*}

where *f* is the volume fraction of the LBPF; *κ* and *μ* are the bulk modulus and shear modulus of the silicone matrix, respectively; *α* is the thermal expansion coefficient of the silicone matrix; and *V*_0_ and *V* are the initial volume and expanded volume of the composites, respectively. Therefore, the volume expansion ratio *V/V*_0_ is:


(4) \begin{eqnarray*}\frac{V}{{{V}_0}} = 1 + 3\left( {\frac{f}{{1 - f}}\frac{{3\kappa + 4\mu }}{{12\kappa \mu }}\Delta P + \alpha \Delta T} \right).\end{eqnarray*}

As shown in Fig. 2C, the volume expansion ratio calculated based on the above theory is in good agreement with the experimental results, proving the rationality of this estimation method. It can also be seen that the volume expansion ratio of the magnetically responsive liquid–vapor phase transition composites can be as high as 2100% at 120°C, indicating its large deformation performance. In addition, as previously mentioned, the expansion of the magnetically responsive liquid–vapor phase transition composites during heating is caused by the internal pressure change of the microchambers caused by the liquid–vapor phase transform of the LBPF. To guide the design process of the PMM, we used the equivalent method to analyse the deformation of the PMM in different states through finite element simulation. As shown in Fig. 2D, the finite element simulation predictions agree well with the experiment results, verifying the accuracy of the models.

In order to illustrate the principle of the shape locking and energy storing of PMMs, we analysed their force situations. As shown in Fig. 2E–H, the shape locking of PMMs is achieved by the friction force *F*_friction_ of the substrate to the PMMs and the magnetic force *F*_magnetism_ on the PMMs under *B*. For the 2D morphing PMM, under the shape-locking state, each of its basic actuating units is subjected to an elastic force *F*_elasticity_ tending to restore the initial flat shape and the applied *B* generates a magnetic force *F*_magnetism_ perpendicular to the working board, which leads to a friction force *F*_friction_ that overcomes *F*_elasticity_; in this way, the locking of bending deformation is realized (Fig. 2E). Under the energy-storing state, each heated basic actuating unit of the PMM is subjected to an actuating force *F*_actuation_ tending to bend and *F*_friction_ caused by *F*_magnetism_ can be used to overcome *F*_actuation_, thus realizing the locking of the initial flat shape and the storage of the elastic potential energy (Fig. 2F). For the 2D-to-3D morphing PMM, under the shape-locking state, the elastic force *F*_elasticity-_*_x_* in the *x* direction is overcome by *F*_friction_, while the elastic force *F*_elasticity-_*_y_* in the *y* direction is overcome by *F*_magnetism_ (Fig. 2G); under the energy-storing state, the actuating force *F*_actuation-_*_x_* in the *x* direction is overcome by *F*_friction_, while the actuating force *F*_actuation-_*_y_* in the *y* direction is overcome by *F*_magnetism_ (Fig. 2H). In this way, the shape locking in working Mode 1 and energy storing in working Mode 2 of PMMs with different deformation types can be achieved. The detailed force analysis can be seen in the Supplementary information.

### Characterization of actuation, shape locking and rapid deformation

**Figure 3.**
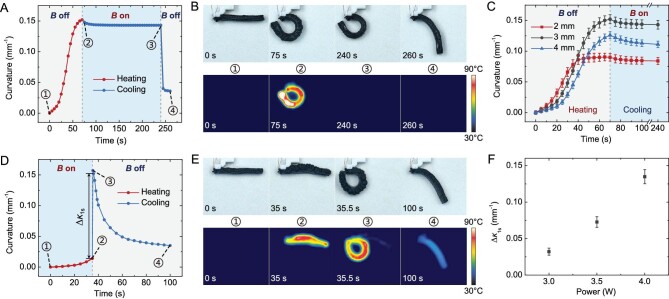
Actuation, shape-locking and rapid deformation characteristics of the basic actuating unit of the PMMs. (A) Time response of the basic actuating unit during the bending–locking–recovery process in working Mode 1. (B) Optical and infrared (IR) image sequences corresponding to the four states in (A). (C) Time responses of basic actuating units with different thicknesses in working Mode 1 (active layer thicknesses of 2, 3 and 4 mm, and strain-limited layer thicknesses of 1 mm). (D) Time response of the basic actuating unit during the energy storage–rapid deformation–recovery process in working Mode 2. (E) Optical and IR image sequences corresponding to the four states in (D). (F) The variation in Δ*κ*_1s_ with different heating powers in working Mode 2 (the heating time during the energy-storage stage here was 35 s). In all experiments carried out above, except for the investigated variable parameters, in (A)–(C) we used an active layer thickness of 3 mm, a strain-limiting layer thickness of 1 mm and a heating power of 3.5 W; in (D)–(F) we used an active layer thickness of 3 mm, a strain-limiting layer thickness of 1 mm and a heating power of 4 W.

To characterize the actuation, shape-locking and rapid deformation properties of PMMs, for the basic actuating unit, we investigated the effect of relevant parameters on the performance. The performance of the basic actuating unit in working Mode 1 is demonstrated in Fig. 3A–C. Figure 3A shows the variation in the curvature of the basic actuating unit with time during the bending–locking–recovery process in working Mode 1. It can be seen that after stopping heating and turning on *B*, the basic actuating unit responded quickly and finally retained most of the bending deformation without additional energy consumption through magnetically assisted shape locking. After *B* was turned off, the basic actuating unit quickly restored most of the deformation retained in the previous stage, while the residual deformation that required longer recovery time was reasonable and acceptable. Structural parameters are important factors that affect the actuating performance. With the same heating power, different layer thicknesses had obvious effects on the response speed, maximum curvature and retained curvature of the basic actuating unit, as shown in Fig. 3C. To characterize the shape-locking capability in working Mode 1, we define the deformation retention ratio in working Mode 1 as:


(5) \begin{eqnarray*}{R}_{\rm{r}} = \frac{{{\kappa }_{\rm{r}}}}{{{\kappa }_{{\rm{max}}}}},\end{eqnarray*}

where *κ*_r_ is the curvature retained after cooling and *κ*_max_ is the maximum curvature achieved before stopping heating. By calculating *R*_r_, we can evaluate the shape-locking effect of the basic actuating unit of the PMMs. Under the experimental conditions studied, the *R*_r_ of the basic actuating unit with an active layer thickness of 2 mm can reach 93.3%, proving that most of the deformation can be retained by adopting the magnetically assisted shape-locking strategy proposed in this work. Figure 3D–F demonstrates the performance of the basic actuating unit in working Mode 2. The variation in the curvature of the basic actuating unit with time during the process of energy storage–rapid deformation–recovery in working Mode 2 is shown in Fig. 3D. During the magnetically assisted energy-storage stage, the curvature did not increase significantly, although the basic actuating unit was heated, while the stored elastic potential energy gradually increased. After *B* was turned off, the basic actuating unit rapidly reached the maximum bending curvature (within 0.5 s in Fig. 3D) due to the release of the elastic potential energy stored during the previous stage. To characterize the rapid deformation property, in working Mode 2, we define the increase in curvature in the 1 s just after *B* was turned off as Δ*κ*_1s_ (Fig. 3D). As shown in Fig. 3F, with the same structural parameters and heating time, different Δ*κ*_1s_ values can be achieved by adjusting the heating power—that is, the basic actuating unit can be controlled to achieve different adjustable deformation states. Therefore, in contrast to previously reported methods based on bistable or multistable structures, which can only switch deformations between a restricted few preset states, the strategy of magnetically assisted energy storage and release proposed in this work not only ensures rapid deformation, but also enables fully adjustable energy-storage and deformation states. In Fig. 3B and E, on the one hand, the friction force of the substrate to the basic actuating unit prevented it from fully recovering its initial flat shape; on the other hand, the residual deformation takes longer to recover further. As shown in Supplementary Fig. S8, the basic actuating unit of the PMMs showed no apparent performance degradation in 10 repeated cycles performed in 1200 s, which is comparable to other state-of-the-art liquid–vapor phase transition composites [20]. In addition, according to some strengthening methods on liquid–vapor phase transition composites [33], after long-term use, PMMs can be immersed in LBPF so that the internal microchambers of the magnetically responsive liquid–vapor phase transition composites can be filled with LBPF again to restore their actuating performance. When comparing Fig. 3A and Supplementary Fig. S8, when *B* was turned off, the sample in Fig. 3A was heated at 3.5 W for 70 s, while the sample in Supplementary Fig. S8 was heated at 3.8 W for 30 s, so the maximum curvature in Fig. 3A was larger than that in Supplementary Fig. S8. In addition, on the one hand, as mentioned above, the friction force of the substrate on the basic actuating unit had a hindering effect on its shape recovery. Unlike the sample in Fig. 3A, which was in contact with the substrate and was affected by the friction force, the sample in Supplementary Fig. S8 had one end fixed to the fixture while operating suspended without contacting with the substrate, thus showing a better recovery performance than the sample in Fig. 3A. On the other hand, due to the relatively small maximum curvature reached during the actuating stage, the sample in Supplementary Fig. S8 recovered its initial state more quickly relative to the sample in Fig. 3A. Referring to the method in [16], we measured the maximum unidirectional blocked force obtained by magnetically responsive liquid–vapor phase transition composites under different blocked strains. For the convenience of measurement and calculation, we used linear actuated samples similar to those in [16]. The measured force-strain characteristics are shown in Supplementary Fig. S9. It can be seen that the blocked force can reach ∼110 N under a zero-strain state and it decreases with the increase in the blocked strain. As shown in Supplementary Fig. S10, using three different substrate conditions of polyimide tape, polyester–cotton cloth and paper tape, we investigated the deformation retention ratio *R*_r_ of the basic actuating unit of the PMMs when operating in working Mode 1 on different substrates. It can be seen from Supplementary Fig. S10 that the substrate condition had a slight effect on *R*_r_, which decreased slightly on the smoother substrate (polyimide tape). However, in the studied range, the *R*_r_ values on all three substrates were >90%, i.e. all had good shape-locking properties and all could meet the requirements of use in this paper. In addition, since the shape locking of PMMs requires the friction force provided by the substrate under the magnetic field, the substrate is necessary to achieve shape locking of PMMs.

### Customizable structures and deformations

**Figure 4.**
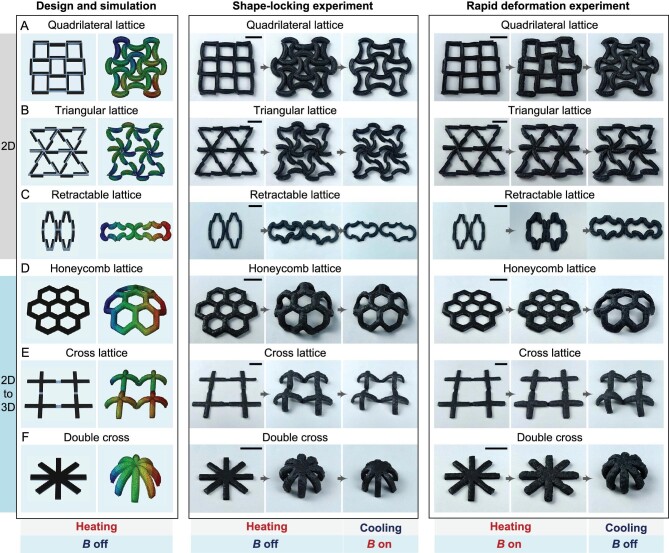
Design, simulation and demonstration of 2D and 2D-to-3D morphing PMMs. Demonstrations include deformation and shape locking of PMMs with different pattern configurations in working Mode 1 and their energy storage and rapid deformation in working Mode 2. The designed patterns of 2D morphing PMMs include (A) a quadrilateral lattice, (B) a triangular lattice and (C) a retractable lattice, and the designed patterns of 2D-to-3D morphing PMMs include (D) a honeycomb lattice, (E) a cross lattice and (F) a double cross. A magnetic field parallel to the normal direction of the PMM plane was applied for magnetically assisted shape locking and energy storage. Scale bars, 2 cm.

As mentioned above, by designing the positional relationship between the PMMs and their basic actuating units, we can control the deformation type of the PMMs to be 2D or 2D-to-3D. Based on this strategy, combined with different pattern designs under the guidance of finite element simulation, we developed a range of PMMs with different customized structures and deformations. As shown in Fig. 4, as demonstrations, we developed three types of 2D morphing PMMs (the quadrilateral lattice, triangular lattice and retractable lattice) and three types of 2D-to-3D morphing PMMs (the honeycomb lattice, cross lattice and double cross), using the fabrication method shown in Supplementary Fig. S3. As shown in Fig. 4, Supplementary Movie S2 and Supplementary Movie S4, in working Mode 1, PMMs with different pattern configurations performed different deformations and shape locking in sequence. As shown in Fig. 4, Supplementary Movie S3 and Supplementary Movie S5, in working Mode 2, these PMMs performed energy storage and different rapid deformations in sequence. Therefore, by arranging a series of bilayer basic actuating units in different customized patterns, the complex and diverse structural forms and deformation types of PMMs can be realized and can be applied in different working modes. In addition, it can be seen that the finite element simulation predictions in Fig. 4 are in good agreement with the experiment results, proving the feasibility of using finite element analysis to simulate the deformation of PMMs so as to guide the design process.

### Function enhancements and applications

**Figure 5.**
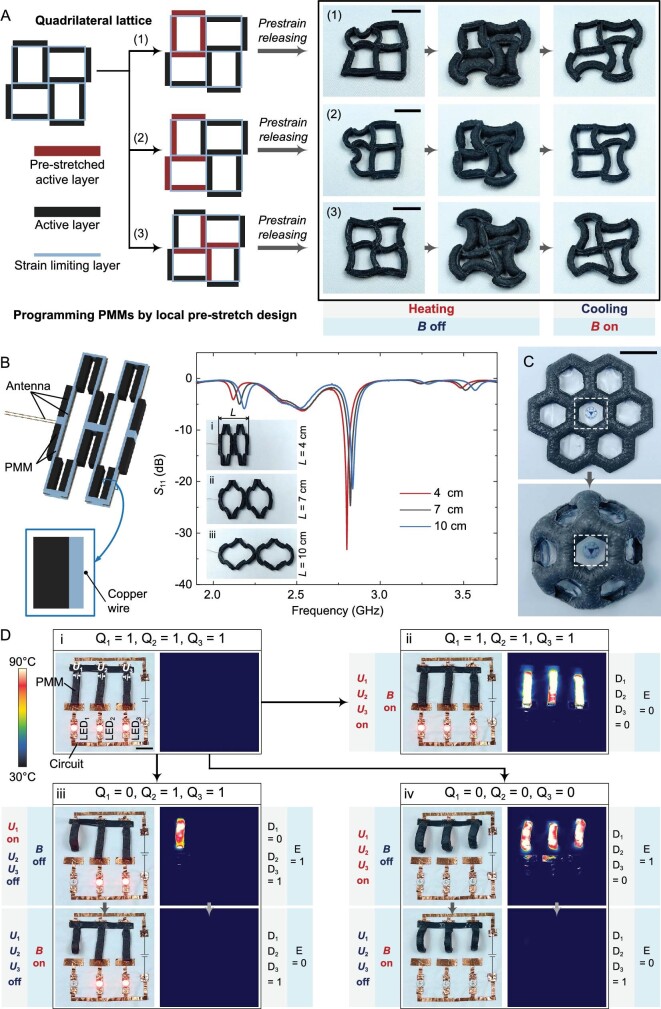
Programmed PMM, reconfigurable antenna, soft lens and soft mechanical memory system built with PMMs. (A) Programming PMMs by local prestretch design. Taking three different local prestretching designs of a quadrilateral lattice as an example. (B) Structural illustration of the reconfigurable antenna and its return loss *S*_11_ characteristic in different shape-locking states. The reconfigurable antenna length *L* was 4, 7  and 10 cm, respectively. (C) Observation ofa logo (emblem of Zhejiang University) through the PMM-based soft lens with tunable imaging. (D) Optical and IR image sequences demonstrating the working process of the soft mechanical memory system that was assembled from PMM-based soft mechanical switches and a flexible circuit. (i) Original information (*Q*_1_, *Q*_2_, *Q*_3_ = 1) can be (ii) retained or rewritten as (iii) new information (*Q*_1_ = 0, *Q*_2_, *Q*_3_ = 1) or (iv) new information (*Q*_1_, *Q*_2_, *Q*_3_ = 0) and then latched. Scale bars, 2 cm.

Enabled by integrating the material system with the lattice metamaterials, different regions of PMMs can be programmed to have different properties. As shown in Fig. 5A, taking the ‘quadrilateral lattice’ type of PMM in Fig. 4A as an example, we demonstrated the capability of PMMs to be programmed through local prestretching. The specific fabrication method is shown in Supplementary Fig. S4. By selectively setting prestretched active layers locally in PMMs, after releasing the prestrain, the basic actuating units in these regions obtain a curved initial shape and the bending curvature can be adjusted by changing the prestrain [34], while the basic actuating units in other regions still maintain the flat initial shape—that is, the programming of the initial shape of the PMMs is realized. When heated, the basic actuating units with a curved initial shape flatten, while the basic actuating units with a flat initial shape bend—that is, the programming of the deformation type of the PMMs is realized. As shown in Fig. 5A and Supplementary Movie S6, through three different local prestretching designs, the quadrilateral lattice can be programmed into different initial shapes and then they can achieve different programmed deformation and shape locking in working Mode 1. In this way, the PMM as a whole can be programmed to have a rich variety of initial shapes and deformation types. This function demonstrates that the lattice metamaterial structure endows PMMs with the ability to be selectively programmed to have different properties in different regions.

Benefitting from the dynamically controllable shape-locking performance of PMMs, as a proof of concept, we developed a reconfigurable antenna with on-demand deformation and shape locking based on the ‘retractable lattice’ type of PMM in Fig. 4C. The ability to change shape and corresponding performance can enable reconfigurable antennas to adapt to different tasks. As shown in Fig. 5B, the reconfigurable antenna consisted of a PMM and copper wires. The copper wire was interspersed and fixed on the strain-limiting layer of the PMM, so it would not affect the normal deformation of the PMM and it could change its shape with the deformation of the PMM. Supplementary Fig. S4 shows the specific fabrication process of the reconfigurable antenna. As shown in Supplementary Movie S7, under working Mode 1, the reconfigurable antenna was gradually unfolded from the folded state when heated and *B* was turned off, and could then be locked in the desired deformed shape when cooled and *B* was turned on. On the basis of the large deformation of the liquid–vapor phase transformation itself, through the pattern configuration design of the PMM, the PMM-based reconfigurable antenna could achieve a more significant deformation, which is beneficial to expand the adjustable range of its performance. As shown in Fig. 5B, by controlling the heating conditions, the reconfigurable antenna can be controlled to reach different deformation states on demand and then be locked through turning on *B*. At the same time, by changing the locked shape of the reconfigurable antenna, its return loss *S*_11_ characteristic can be tuned. Therefore, we achieved controllable on-demand deformation, shape locking without energy consumption and antenna performance tuning of a reconfigurable antenna based on the PMM.

2D-to-3D morphing PMMs can realize controllable deformation from a 2D plane to complex 3D curved surfaces, which is suitable for applications in tunable optics. By combining the ‘honeycomb lattice’ type of PMM in Fig. 4D with soft convex lenses based on polydimethylsiloxane (PDMS) membranes and silicone oil, we developed a PMM-based soft lens with tunable imaging and its specific fabrication process is shown in Supplementary Fig. S4. As shown in Fig. 5C and Supplementary Movie S8, when observing a logo through the soft lens, as the PMM was heated and deformed into a 3D curved surface, the distance between the central soft convex lens and the logo gradually increased and the observed logo image was gradually enlarged by ∼143%. This magnification effect is based on the principle similar to the magnification of a magnifying glass in the range of 1 focal length. This demonstration illustrates the application potential of the complex deformation capabilities endowed by metamaterial structures to PMMs.

Mechanical memory systems constructed from smart soft materials have the potential to be applied in intelligent mechanical systems integrated with mechanical computing [35–39]. Taking advantage of the reversible active deformation and dynamically controllable shape-locking capabilities of a PMM, as a proof of concept, here we demonstrated a soft mechanical memory system based on a 2D-to-3D morphing PMM, which is a kind of transformation of ‘cross lattice’ and ‘double cross’ PMMs as shown in Fig. 4E and F. As shown in Supplementary Fig. S6A, the soft mechanical memory system was assembled by using PMM-based soft mechanical switches and a flexible circuit composed of copper foil tapes and LEDs. The soft mechanical switches based on the PMM consisted of active layers embedded with constantan wire, a strain-limiting layer and copper foil tapes, which were bonded in sequence. The specific fabrication of the soft mechanical memory system is shown in Supplementary Fig. S4. The information storage of the soft mechanical memory system was based on the principle of the D latch (Supplementary Fig. S6B). Each basic actuating unit in the PMM-based soft mechanical switches can be viewed as a D latch that can be used to store 1 bit of binary information. As shown in Supplementary Fig. S6D, in such a D latch, the magnetic field (*B*) was used as input *E*, the voltage (*U*) for Joule heating was used as input *D* and the LED was used as output *Q*. According to the truth table of the D latch (Supplementary Fig. S6C), when *E* = 0, *Q* is latched in the original state without being affected by *D*, thus realizing the storage of information, which corresponds to that when *B* is turned on, and the shape of the soft mechanical switch is locked; when *E* = 1, *Q* is determined by *D*, thereby realizing the rewriting of information, which corresponds to that when *B* is turned off, and the state of the soft mechanical switch is determined by whether *U* is applied. Since each basic actuating unit can be individually controlled by applying *U*, theoretically, the PMM-based soft mechanical memory system containing *n* basic actuating units can store *n* bits of binary information. To illustrate the above capabilities, we used a soft mechanical memory system that can store 3 bits of binary information as a demonstration. As shown in Fig. 5D and Supplementary Movie S9, each bit of information can be latched or rewritten by controlling *B* and *U*. For each D latch with original information (*Q* = 1), when turning on *B* (*E* = 0), even if turning on *U* to heat (*D* = 0), the soft mechanical switch was still locked in the original closed state, thus the D latch could keep the original information (*Q* = 1); under working Mode 1, by first turning off *B* (*E* = 1) and turning on *U* (*D* = 0) to disconnect the soft mechanical switch and then turning on *B* (*E* = 0) and turning off *U* (*D* = 1) to lock the soft mechanical switch in the disconnected state, the D latch can realize rewriting new information (*Q* = 0) and latching it, respectively. By performing the above operations on each bit in the multi-bit soft mechanical memory system, the storage and rewriting of multi-bit binary information can be realized. Therefore, we demonstrated the feasibility of developing soft mechanical memory systems utilizing PMMs with reversible active deformation and dynamically controllable shape-locking capabilities.

**Figure 6.**
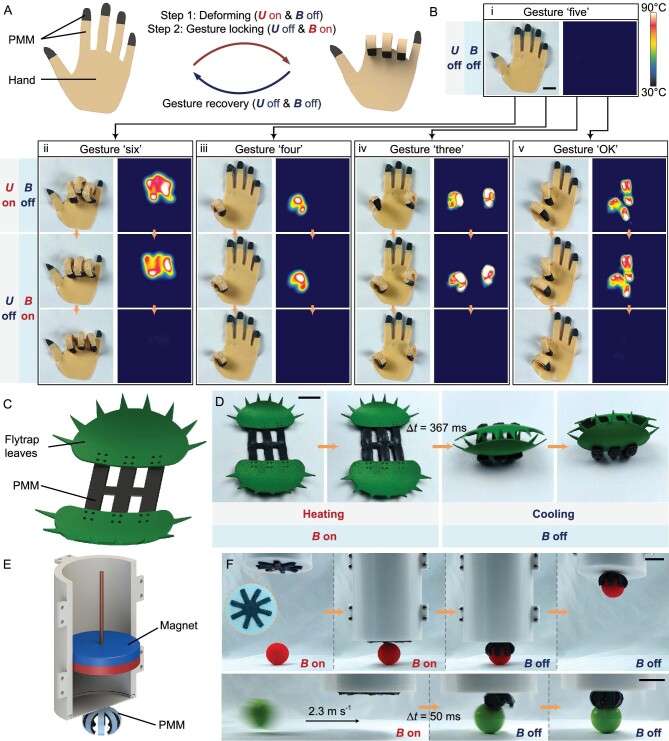
Biomimetic hand, flytrap and self-contained soft gripper system built with PMMs. (A) Schematic illustration of the deformation, gesture locking and gesture recovery of the biomimetic hand in working Mode 1. (B) Optical and IR image sequences demonstrating programmable deformation and gesture locking of the biomimetic hand. The biomimetic hand can change from the initial gesture (i) ‘five’ to many different gestures including (ii) ‘six’, (iii) ‘four’, (iv) ‘three’ and (v) ‘OK’. (C) Schematic illustration of the structural composition of the biomimetic flytrap. (D) Demonstration of magnetically assisted energy storage and rapid closure of the biomimetic flytrap under working Mode 2. (E) Schematic illustration of the structural composition of the self-contained PMM-based soft gripper system. (F) Demonstration of the soft gripper grasping and lifting a ball, and a demonstration of the soft gripper responding quickly within 50 ms to stop a fast-moving ball. Scale bars, 2 cm.

PMMs can also achieve programmable deformation. Soft robotics have unique advantages in information expression and communication; as a demonstration, here a type of biomimetic hand with programmable deformation and gesture locking based on a 2D-to-3D morphing PMM was developed, which is a kind of transformation of ‘cross lattice’ and ‘double cross’ PMMs as shown in Fig. 4E and F. The biomimetic hand was composed of a strain-limiting layer and active layers embedded with constantan wires. The fingertips of both layers contained CIP and thus had magnetically responsive characteristics. Supplementary Fig. S5 shows the specific fabrication process of the biomimetic hand. As shown in Fig. 6A, under working Mode 1, the biomimetic hand can achieve the desired deformation and gesture locking in sequence, and the gesture can be recovered after cooling and turning off *B*, reflecting the reversible active deformation and shape locking of PMMs. In the biomimetic hand, five independently controlled basic actuating units were used as fingers. Therefore, by controlling the combination of activated basic actuating units, the deformation of the biomimetic hand can be programmed into different gestures. Using the above method, as shown in Fig. 6B and Supplementary Movie S10, the biomimetic hand can programmably change from the initial gesture ‘five’ to a variety of different gestures including ‘six’, ‘four’, ‘three’ and ‘OK’, and can lock gestures without energy consumption, proving the rich and programmable morphing forms and the gesture-locking property of the PMM-based biomimetic hand. The above process mimicked the behavior of the human hand making various gestures to convey information for communication.

In nature, Venus flytraps rapidly close their leaves by abruptly releasing stored elastic potential energy and achieve the fastest motion response in the plant kingdom [30]. Similarly, salamanders quickly launch their tongues to grab prey by releasing stored elastic potential energy [31]. Drawing on the wisdom of natural creatures, in working Mode 2, the elastic energy-storage performance of the PMM endows it with the ability to deform rapidly, thus overcoming the limitation of the relatively slow response speed of the traditional liquid–vapor phase transform actuating method. To demonstrate this property of PMMs, we developed a biomimetic flytrap with rapid response based on a 2D-to-3D morphing PMM, which is a kind of transformation of ‘cross lattice’ and ‘double cross’ PMMs in Fig. 4E and F. As shown in Fig. 6C, the biomimetic flytrap consisted of a PMM and nylon flytrap leaves. Supplementary Fig. S5 shows the specific manufacturing process of the biomimetic flytrap. Under working Mode 2, as shown in Fig. 6D and Supplementary Movie S11, first when heating and *B* was turning on, the biomimetic flytrap gradually stored energy, then when heating was stopped and *B* was turned off, the biomimetic flytrap rapidly released the elastic potential energy stored during the previous stage to rapidly close the leaves and the closing response time Δ*t* could reach 367 ms. The above process mimicked the natural behavior of the Venus flytrap of rapidly closing its leaves to catch prey by releasing stored elastic potential energy. Compared with previous reports on soft actuators based on liquid–vapor phase transformation [16–20,40–43], the PMM-based biomimetic flytrap developed in this work improved the deformation response speed by at least an order of magnitude by virtue of a magnetically assisted energy-storage and energy-release strategy, proving that PMM has the ability to achieve controllable and rapid response based on energy storage.

To demonstrate that the utility of the material system is not affected by the magnetic device, we developed a demonstration of a self-contained PMM-based soft gripper system, as shown in Fig. 6E, which included a ‘double cross’ type of PMM with prestretching as the soft gripper and a magnet providing the *B*. Its specific fabrication process and working process can be seen in Supplementary Fig. S5 and the Supplementary information. As shown in Fig. 6F and Supplementary Movie S12, the soft gripper can move position to grasp or release objects and can respond quickly to stop a fast-moving ball. The magnet-based device that provides *B* for the PMM was relatively small and compact. This demonstration proves that the magnetic device does not compromise the portability and utility of the PMM-based soft gripper system and also proves the fast response capability of the soft gripper. General magnetic actuated soft robots require complex external devices to provide magnetic fields with adjustable direction and strength, which affects their miniaturization and portability. PMMs are mainly actuated by phase transition and the auxiliary magnetic device is relatively simple. The demonstrated soft gripper system including magnets can be easily moved, which proves the advantages of PMMs over general magnetic actuated soft robots in terms of miniaturization and portability. In addition, general magnetic actuated soft robots need to be magnetized to orient the magnetic domains during fabrication, while PMMs do not need this step, which illustrates the advantages of PMMs over general magnetic actuated soft robots in terms of fabrication simplicity.

## DISCUSSION

We have introduced a class of phase-transforming mechanical metamaterials that combines the advantages of existing liquid–vapor phase transition composites and active mechanical metamaterials, and integrates dynamically controllable shape-locking capabilities. A series of bilayer basic actuating units were periodically arranged to form the PMMs. Complex and diverse structures and deformations could be customized by controlling the pattern configuration of the PMMs and the positional relationship between the PMMs and their basic actuating units. The design process of PMMs was guided by theoretical analysis and finite element simulation. The applied magnetically responsive liquid–vapor phase transition composites endowed the PMMs with active reversible large deformation and stretchability. The added CIP endowed the PMMs with dynamically controllable shape-locking performance, thereby achieving magnetically assisted shape-locking and energy-storage functions under different working modes. The rapid reversible shape-locking feature eliminated the constant energy consumption required to maintain the actuated shape. Also, the adjustable energy storage and release facilitated rapid and adjustable deformation, which overcame the limitation of relatively slow response speed of traditional liquid–vapor phase transition actuating methods. Using PMMs, a range of function enhancements and applications were demonstrated, including programmed PMM by local prestretch design, reconfigurable antenna with on-demand deformation and shape locking, soft lens with tunable imaging, soft mechanical memory, biomimetic hand with programmable deformation and gesture locking, biomimetic flytrap with rapid response and self-contained soft gripper with rapid response.

Relative to active mechanical metamaterials using smart soft materials with different actuation technologies such as shape memory polymers [5–7], hydrogels [8,9], soft pneumatic actuators [10,11] and magnetically actuated soft materials [12–15], liquid–vapor phase transition composites [16–20] with high stretchability, active reversible large volume change, no need for external gas sources or aqueous medium, and simple and convenient fabrication methods is expected to be used in the development of active metamaterials with richer properties, functions and applications, but this exploration is limited by their relatively simple and restricted structural forms and deformation types. Also, liquid–vapor phase transition composites are hindered by the need for continuous energy consumption to maintain the actuated shape and the relatively slow response speed. Shape locking of active mechanical metamaterials can eliminate continuous energy consumption, but existing methods utilizing material stiffness changes at different temperatures [5,7,27–29] suffer from problems of slow locking or unlocking speed, lack of active reversible deformation and stretchability. Energy storage is expected to be used to improve the response speed of active mechanical metamaterials based on liquid–vapor phase transition composites, but existing methods for energy storage based on bistable or multistable structures in flexible mechanical metamaterials [25,26,32] are restricted by a limited few preset deformation states and the lack of active reversible deformation. As indicated in Supplementary Table S2, compared with previously reported liquid–vapor phase transition composites, active mechanical metamaterials with shape locking and flexible mechanical metamaterials with energy storage, the PMMs reported here integrate many advantages in a single metamaterial system, including customizable complex structures and deformations, active reversible large deformation, rapid reversible shape locking, rapid and adjustable deformation based on energy storage and release, and stretchability.

Although PMMs have many valuable properties, there are still some limitations that need to be further improved. The actuating method of PMMs can be further enriched; for example, the CIPs contained in PMMs have the ability to be magnetically heated under a high-frequency alternating magnetic field due to the hysteresis loss heating mechanism [44], thereby causing the liquid–vapor phase transformation of the contained LBPF. Also, by integrating different kinds of flexible electronic components [23,24], PMMs can realize more abundant functions such as environmental sensing, self-state sensing and wireless communication.

## METHODS

### Materials

The basic actuating units used to construct PMMs consist of two components: the active layer and the strain-limiting layer. The magnetically responsive liquid–vapor phase transition composites for the active layer were prepared by thoroughly mixing CIP (5 μm, Hunan Bohai Advanced Material Technology, China), ethanol (10009218, Sinopharm Chemical Reagent, China), Novec 7000 (3 M, USA) and 00-35 silicone (Ecoflex 00-35 fast, Smooth-on, USA). The material of the magnetically responsive strain-limiting layer was prepared by mixing the components A and B of 30A silicone (PS6600, Shenzhen Yipin Trading, China) in equal proportions and then adding CIP and mixing them uniformly. More details on materials, fabrication and characterization of PMMs can be found in the Supplementary information.

### Theory and simulation of deformation

The saturated vapor pressure properties of ethanol [45] and Novec 7000 [46] are given in Supplementary Fig. S7 and were used to calculate the saturated vapor pressure of the LBPF used in this paper shown in Fig. 2B according to Equation (2). The bulk modulus *κ* = 0.423 MPa [19] and shear modulus *μ* = 0.0216 MPa [47] and thermal expansion coefficient *α* = 284.2 ppm/°C [48] of the silicone matrix were used to calculate the volume expansion ratio *V/V*_0_ of the magnetically responsive liquid–vapor phase transition composites shown in Fig. 2C according to Equation (4). Finite element simulations using ABAQUS were performed to predict the deformation of the PMMs, so as to guide their design. The pressure provided by the liquid–vapor phase transformation of the LBPF was applied to the active layers of the PMMs by the equivalent means—that is, in ABAQUS, the pressure was applied as a load on the inner surfaces of the chambers in the active layers of the PMMs. The tensile tests were performed using an Instron (345 C-05) universal test machine at a speed of 100 mm/min to obtain the material properties of the CIP/LBPF/00-35 silicone composites for the active layer and the CIP/30A silicone composites for the strain-limiting layer shown in Supplementary Fig. S11. The hyperelastic incompressible Yeoh material model [49], in which the strain energy *W* = *C*_10_(*I*_1_−3) + *C*_20_(*I*_1_−3)^2^ + *C*_30_(*I*_1_−3)^3^, was applied to describe the non-linear material behavior of both the CIP/LBPF/00-35 silicone composites and the CIP/30A silicone composites. With the fitting method, the obtained material coefficients for the CIP/LBPF/00-35 silicone composites were *C*_10_ = 0.007 MPa, *C*_20_ = −0.001 MPa and *C*_30_ = 0.00007 MPa, and the obtained material coefficients for the CIP/30A silicone composites were *C*_10_ = 0.08 MPa, *C*_20_ = 0.007 MPa and *C*_30_ = −0.00063 MPa. The results of the finite element simulation analysis of several PMMs with different pattern configurations are shown in Figs 2D and 4. It can be seen that the finite element simulation predictions in Figs 2D and 4 are in good agreement with the experiment results, demonstrating that our material deformation models are reliable and correct.

## Supplementary Material

nwad192_Supplemental_Files
