# Supplementary material for: Phase-transforming mechanical metamaterials with dynamically controllable shape-locking performance
Source: Natl Sci Rev. 2023 Jul 8;10(9):nwad192. doi: 10.1093/nsr/nwad192 (PMC10411672; doi:10.1093/nsr/nwad192)
Supplement: nwad192_Supplemental_Files [file nwad192_supplemental_files.zip › Supplementary data.pdf]

Supplementary Information

Phase-transforming mechanical metamaterials with dynamically controllable shape-locking performance

Yiding Zhong et al.

Corresponding author. Wei Tang: weitang@zju.edu.cn; Jun Zou: junzou@zju.edu.cn

This PDF file includes:

Methods
Figure S1. Confocal laser microscope image of magnetically responsive liquid-vapor phase transition composites.
Figure S2. Demonstration of stretchability of PMMs.
Figure S3. Fabrication process of PMMs.
Figure S4. Fabrication process of (A) programmed PMM, (B) reconfigurable antenna, (C) soft lens and (D) soft mechanical memory.
Figure S5. Fabrication process of (A) biomimetic hand, (B) biomimetic flytrap and (C) self-contained soft gripper.
Figure S6. Information storage principle of soft mechanical storage system.
Figure S7. The saturated vapor pressure properties of ethanol and Novec 7000.
Figure S8. Cyclic bending-recovery process of the basic actuating unit of the PMMs in working mode 1.
Figure S9. Blocked force at various elongations for constrained cylindrical actuation of magnetically responsive liquid-vapor phase transition composites in PMMs.
Figure S10. The effect of different substrate conditions on the deformation retention ratio R_r of the basic actuating unit of the PMMs.
Figure S11. The stress-extension ratio relationships of the CIP/LBPF/00-35 silicone composites and the CIP/30A silicone composites in tensile tests.
Table S1. Definition of forces in the force analysis of PMMs.
Table S2. Comparison of PMMs, existing liquid-vapor phase transition composites, active mechanical metamaterials with shape locking, and flexible mechanical metamaterials with energy storage.

Methods

Materials

The basic actuating units used to construct PMMs consist of two components, the active layer and the strain limiting layer. The magnetically responsive liquid-vapor phase transition composites for the active layer was prepared by thoroughly mixing CIP (5 μm, Hunan Bohai Advanced Material Technology, China), ethanol (10009218, Sinopharm Chemical Reagent, China), Novec 7000 (3M, USA), and 00-35 silicone (Ecoflex 00-35 fast, Smooth-on, USA). First, CIP (100 wt%), ethanol (50 vol%), and Novec 7000 (50 vol%) were added to component A of 00-35 silicone and mixed well, then added component B of 00-35 silicone, after fully mixing, the material could be poured into the mold for curing. The mass ratio used here represents $m_{\mathrm{CIP}}/m_{\mathrm{silicone}}$, and the volume ratio used here represents $V_{\mathrm{LBPF}}/V_{\mathrm{silicone}}$, while ethanol and Novec 7000 were used as LBPF. The material of the magnetically responsive strain limiting layer was prepared by mixing the components A and B of 30A silicone (PS6600, Shenzhen Yipin Trading, China) in equal proportions and then adding CIP (100 wt%) and mixing them uniformly. The material could be cast after vacuuming for about 3 minutes.

Fabrication of PMMs and applications

To fabricate the PMMs, as shown in Fig. S3, the first step was to cast uncured CIP/LBPF/00-35 silicone composites, i.e., magnetically responsive liquid-vapor phase transition composites, in the molds to make the active layers. The second step was to cast the uncured CIP/30A silicone composites in the mold to make the strain limiting layer. The third step was to bond the two layers according to the designed pattern and positional relationship, and finally PMMs with customizable structures and deformations could be obtained. As shown in Fig. S4A, programmed PMMs were fabricated similarly to other PMMs, but the active layers at the designed positions needed to be pre-stretched before bonding with the strain limiting layer. As shown in Fig. S4B, the reconfigurable antenna was fabricated in a way similar to that of 2D morphing PMMs, but copper wires were interspersed in the strain limiting layer before bonding the two layers. Two copper wires with a length of 20 cm and a diameter of 0.17 mm were symmetrically distributed in the strain limiting layer, and one of them was used for the return loss S_{11} characteristic test. As shown in Fig. S4C, the PMM-based soft lens was fabricated by bonding a "honeycomb lattice" type PMM with soft convex lenses. The soft convex lens was obtained by bonding two PDMS membranes (thickness 150 μm), injecting silicone oil and sealing. As shown in Fig. S4D, the soft mechanical switches were fabricated in a way similar to that of 2D to 3D morphing PMMs, but the strain limiting layer was unidirectionally pre-stretched with 11% pre-strain and fixed on the fixture before bonding the two layers. After bonding the two layers and releasing the pre-strain, soft mechanical switches with initial bending states were obtained to ensure their close contact with the copper foil tape in the flexible circuit. And helical constantan wires were embedded in the active layers, and copper foil tapes were bonded on the strain limiting layer. The helical constantan wires were obtained by manually winding constantan wires (0.1 mm in diameter) on a stainless steel shaft. And the soft mechanical memory system was fabricated by assembling soft mechanical switches and a flexible circuit consisting of copper foil tapes and LEDs. As shown in Fig. S5A, the biomimetic hand was fabricated in a way similar to that of 2D to 3D morphing PMMs, but the active layer molds were pre-placed with helical constantan wires before casting for subsequent use of Joule heating. And both the active layers and the strain limiting layer realized the selective distribution of CIP at the fingertip by casting different materials in two steps. As shown in Fig. S5B, the biomimetic flytrap was fabricated in a way similar to that of 2D to 3D morphing PMMs, and was connecting to nylon flytrap leaves using transparent nylon threads. As shown in Fig. S5C, the self-contained soft gripper was assembled by a "double cross" type PMM with

pre-stretching, a magnet, and a shell. Among them, the "double cross" type PMM with pre-stretching was fabricated in a way similar to that of 2D to 3D morphing PMMs, but the active layer was pre-stretched before the bonding two layers.

Characterization

The microstructure of the magnetically responsive liquid-vapor phase transition composites was observed and photographed by a confocal laser microscope (VK-X100, Keyence, Japan). In order to measure the volume expansion ratio, each spherical sample of the magnetically responsive liquid-vapor phase transition composites was heated in a silicone oil bath, while being photographed by a digital camera, and the volume of the sample at a certain temperature was obtained by analyzing the captured images. The temperature of the silicone oil bath was controlled by a constant temperature heating pot (DF-101S, Shanghai Lichen Bangxi Instrument Technology, China). Three sets of data were used to calculate the reported volume expansion ratio values. Demonstrations of 2D and 2D to 3D morphing PMMs, programmed PMM, reconfigurable antennas, soft lens, and biomimetic flytrap were actuated by heating with a heat gun. The basic actuating units for performance characterization, the soft mechanical memory and the biomimetic hand were actuated using Joule heating of helical constantan wires embedded in the active layer. When using Joule heating, the heating of a specific area can be controlled by designing the placement of the constantan wires, while the heating temperature can be controlled by adjusting the heating power and heating time under defined experimental conditions. And heating with a heat gun is suitable for occasions that do not require precise control of the heating area or temperature. DC power supplies (MS305D, Dongguan Maisheng Power Technology, China) were used to power the Joule heating. A digital camera and an IR camera (A615, FLIR, USA) were simultaneously used to observe the basic actuating units for performance characterization, the soft mechanical memory and the biomimetic hand, to simultaneously obtain changes in their shape and temperature. The varying curvatures of the basic actuating units were obtained by analyzing images captured by a digital camera using ImageJ. Five sets of data were used to calculate the reported curvature values. A vector network analyzer (LiteVNA) was used to measure the return loss S_{11} characteristic of the reconfigurable antenna. A magnet array consisting of 78 square NbFeB magnets (20 mm × 20 mm × 10 mm) fixed to the lifting platform was used to provide B for shape locking and energy storage. And the on and off states of B were controlled by adjusting the position of the lifting platform to which the magnet array was fixed. And a cylindrical NdFeB magnet (diameter 60 mm, thickness 15 mm) was used in the demonstration of the PMM-based soft gripper.

Self-contained PMM-based soft gripper system

As shown in Fig. S5C, the soft gripper system included a "double cross" type PMM with pre-stretching as the soft gripper, a magnet providing the B and a shell. The B provided by the magnet was used to control the opening and closing of the gripper. And the on and off states of B were controlled by adjusting the position of the magnet in the shell. When B is turned off, the pre-stretched soft gripper is in a bent closed state; when B is turned on, the gripper turns to an open state. Due to the elastic potential energy stored in the open state, the gripper can respond quickly when B is turned off. In future studies, by selectively Joule-heating some fingers in the soft gripper, only the non-heated fingers will bend when B is turned off, thus programming the deformed state of the gripper.

Force analysis of PMMs under shape-locking and energy-storing states

Using the definitions of the relevant forces in Table S1 and the direction of the coordinate system in Fig. 2G, we analyze the force states of the PMMs in Fig. 2E-H.

As shown in Fig. 2E, under shape-locking state, at the cross section taken in the figure, the 2D morphing PMM is in force equilibrium in the x and y directions, respectively, so we have:

$$\sum F_x = F_{\text{friction}} - F_{\text{elasticity}} = 0 \tag{S1}$$

$$\sum F_y = F_{\text{support}} - F_{\text{magnetism}} - G = 0 \tag{S2}$$

In the x direction, F_{friction} is balanced with $F_{\text{elasticity}}$, thus locking the deformation.

As shown in Fig. 2F, under energy-storing state, at the cross section taken in the figure, the 2D morphing PMM is in force equilibrium in the x and y directions, respectively, so we have:

$$\sum F_x = F_{\text{actuation}} - F_{\text{friction}} = 0 \tag{S3}$$

$$\sum F_y = F_{\text{support}} - F_{\text{magnetism}} - G = 0 \tag{S4}$$

In the x direction, F_{friction} is balanced with $F_{\text{actuation}}$, so as to lock in the initial shape and store elastic potential energy.

As shown in Fig. 2G, under shape-locking state, at the cross section taken in the figure, the 2D to 3D morphing PMM is in force equilibrium in the x and y directions, respectively, so we have:

$$\sum F_x = F_{\text{elasticity-}x} - F_{\text{friction}} = 0 \tag{S5}$$

$$\sum F_y = F_{\text{support}} + F_{\text{elasticity-}y} - F_{\text{magnetism}} - G = 0 \tag{S6}$$

In the x direction, F_{friction} is balanced with $F_{\text{elasticity-}x}$; in the y direction, since G is small relative to $F_{\text{magnetism}}$, $F_{\text{elasticity-}y}$ is mainly overcome by $F_{\text{magnetism}}$, thereby achieving locking the deformation.

As shown in Fig. 2H, under energy-storing state, at the cross section taken in the figure, the 2D to 3D morphing PMM is in force equilibrium in the x and y directions, respectively, so we have:

$$\sum F_x = F_{\text{friction}} - F_{\text{actuation-}x} = 0 \tag{S7}$$

$$\sum F_y = F_{\text{support}} + F_{\text{actuation-}y} - F_{\text{magnetism}} - G = 0 \tag{S8}$$

In the x direction, F_{friction} is balanced with $F_{\text{actuation-}x}$, in the y direction, since G is small relative to $F_{\text{magnetism}}$, $F_{\text{actuation-}y}$ is mainly overcome by $F_{\text{magnetism}}$, thereby achieving locking in the initial shape and storing elastic potential energy.

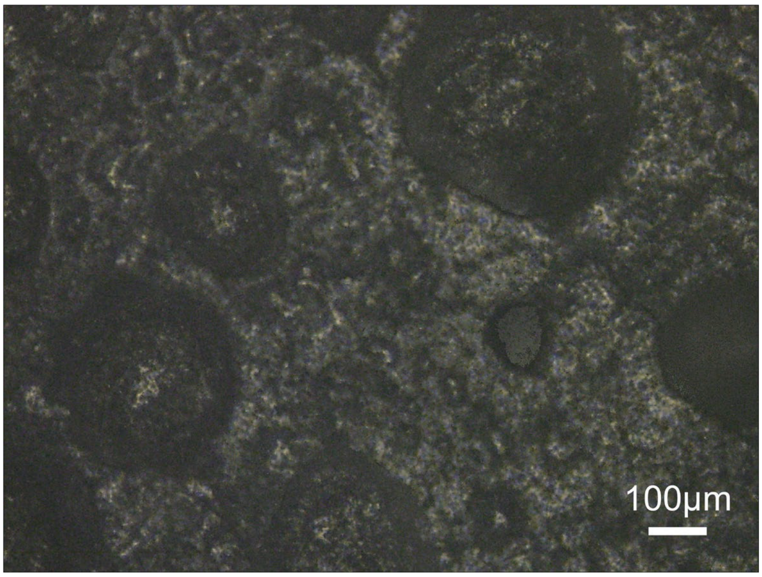

Figure S1. Confocal laser microscope image of magnetically responsive liquid-vapor phase transition composites.

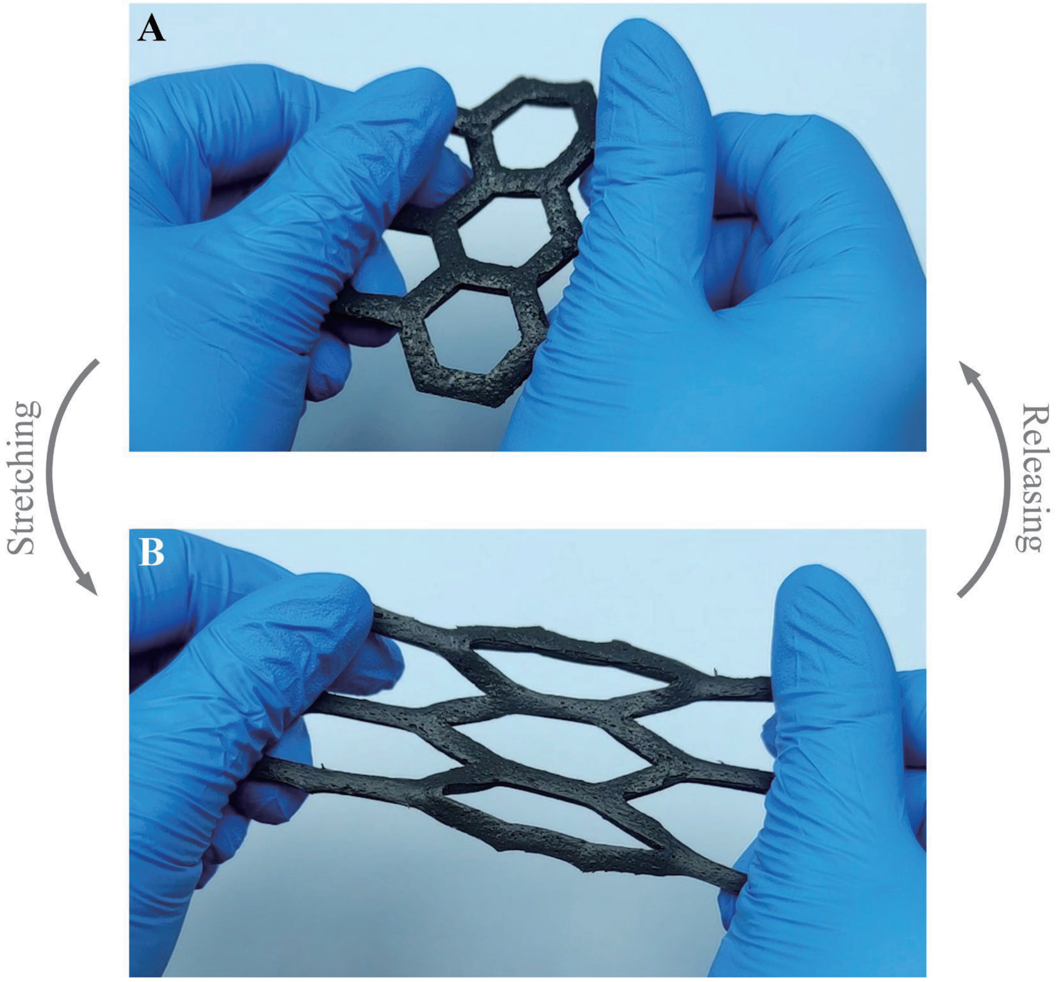

Figure S2. Demonstration of stretchability of PMMs. (A) Initial shape of the PMM in relaxed state. (B) Shape change of the PMM in stretched state.

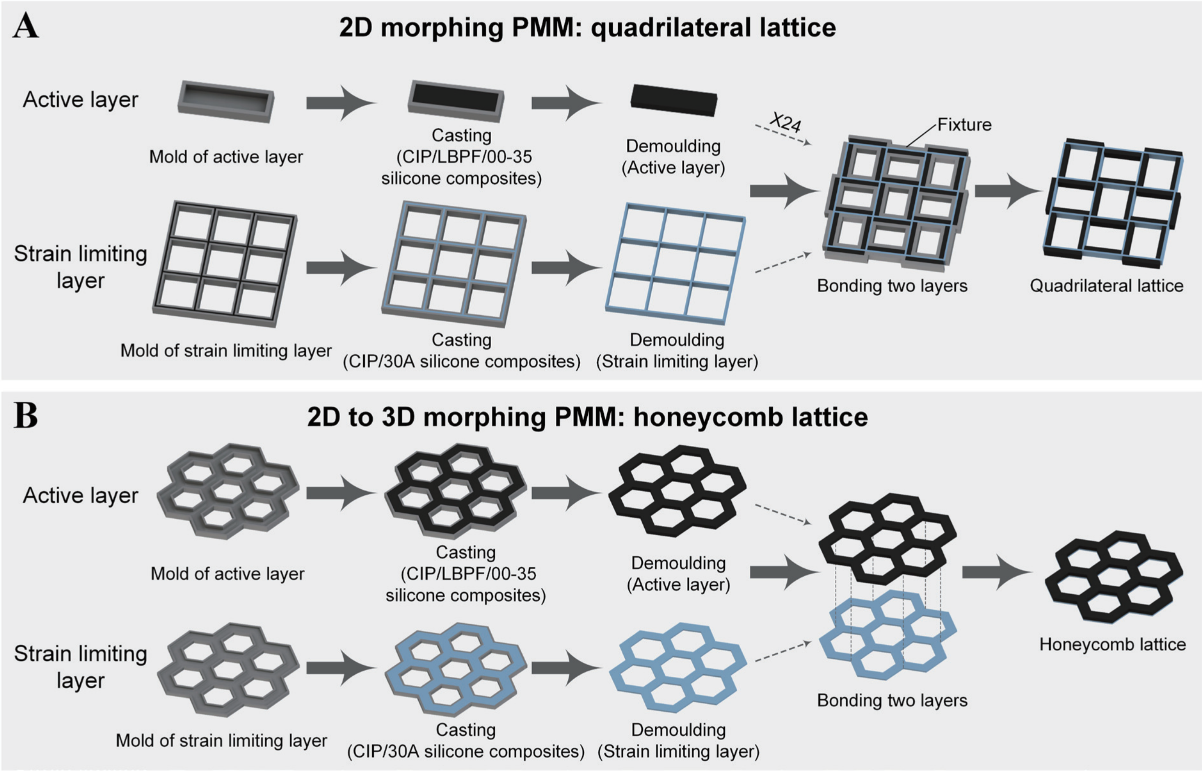

Figure S3. Fabrication process of PMMs. (A) Fabrication of 2D morphing PMMs, taking the quadrilateral lattice as an example. (B) Fabrication of 2D to 3D morphing PMMs, taking the honeycomb lattice as an example.

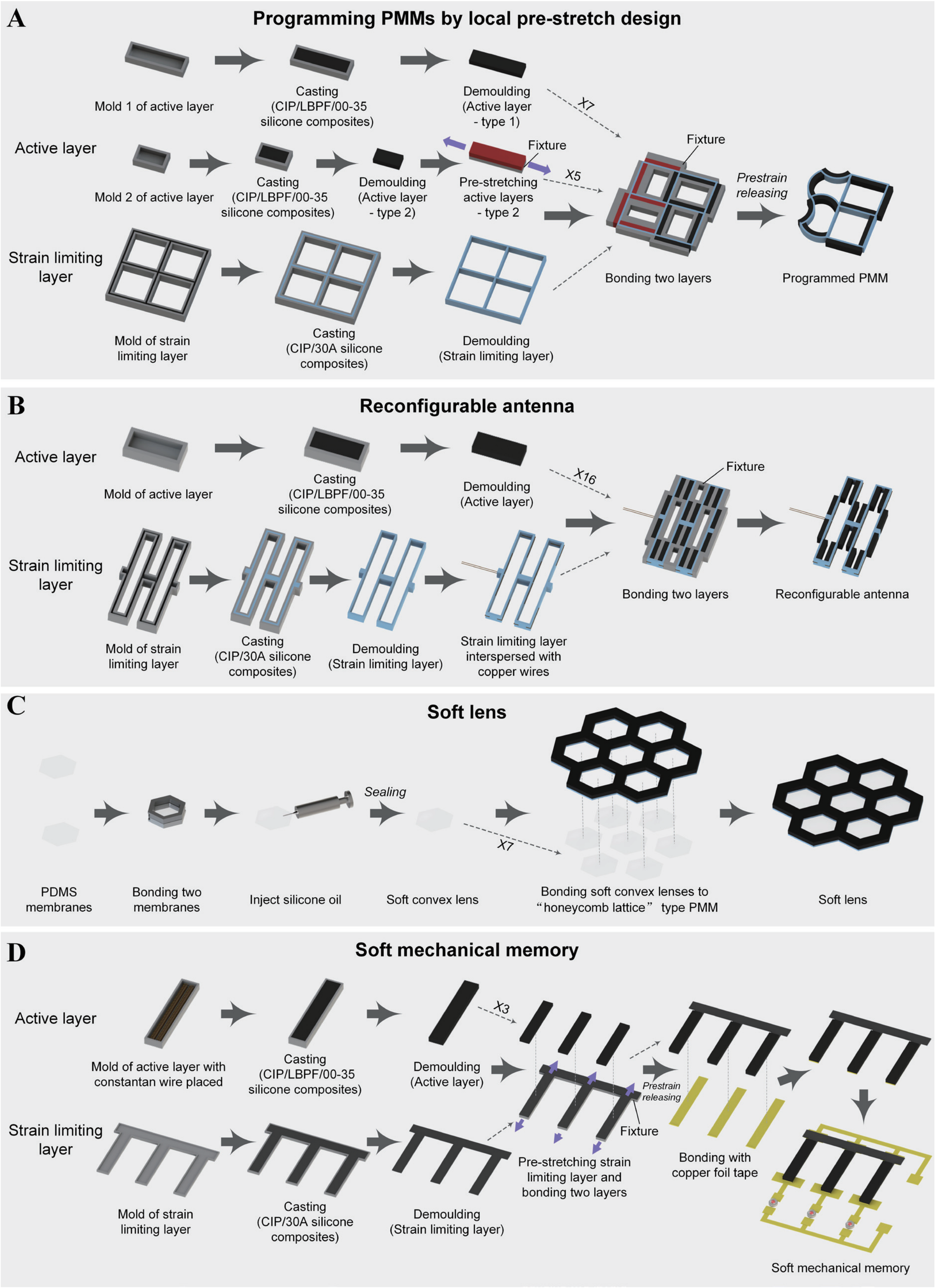

Figure S4. Fabrication process of (A) programmed PMM, (B) reconfigurable antenna, (C) soft lens and (D) soft mechanical memory.

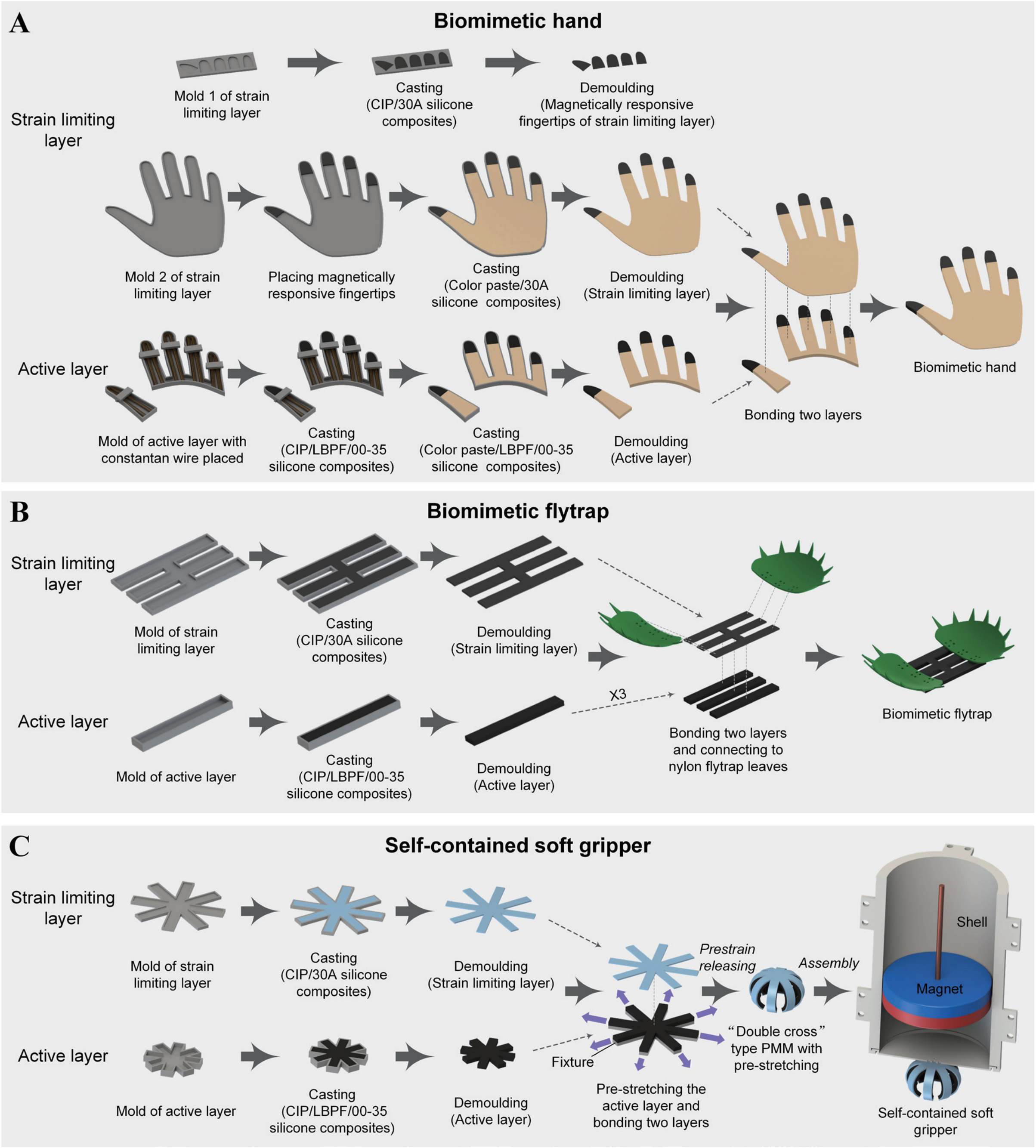

Figure S5. Fabrication process of (A) biomimetic hand, (B) biomimetic flytrap and (C) self-contained soft gripper.

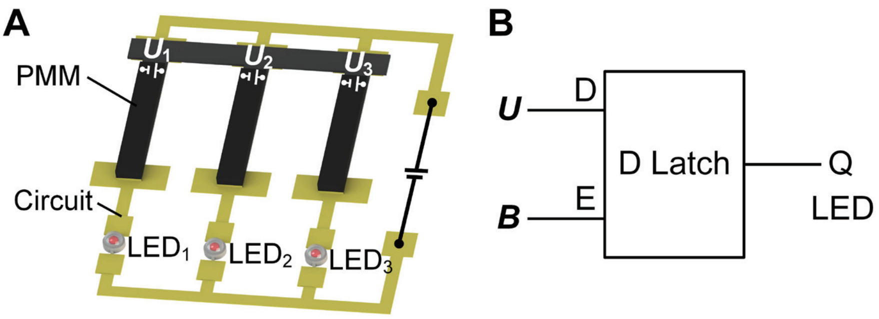

C

E	D	Q
0	0	Latch
0	1	Latch
1	0	0
1	1	1

D

	0	1
E	***B*** on	***B*** off
D	***U*** on	***U*** off
Q	LED off	LED on

Figure S6. Information storage principle of soft mechanical storage system. (A) Schematic diagram of the structural composition of the soft mechanical memory system, which was assembled from PMM-based soft mechanical switches and a flexible circuit. (B) Schematic diagram of the D latch, with *B* as input E, *U* as input D, and LED as output Q, respectively. (C) Truth table for the D latch. (D) Definition of the input and output states of the D latch.

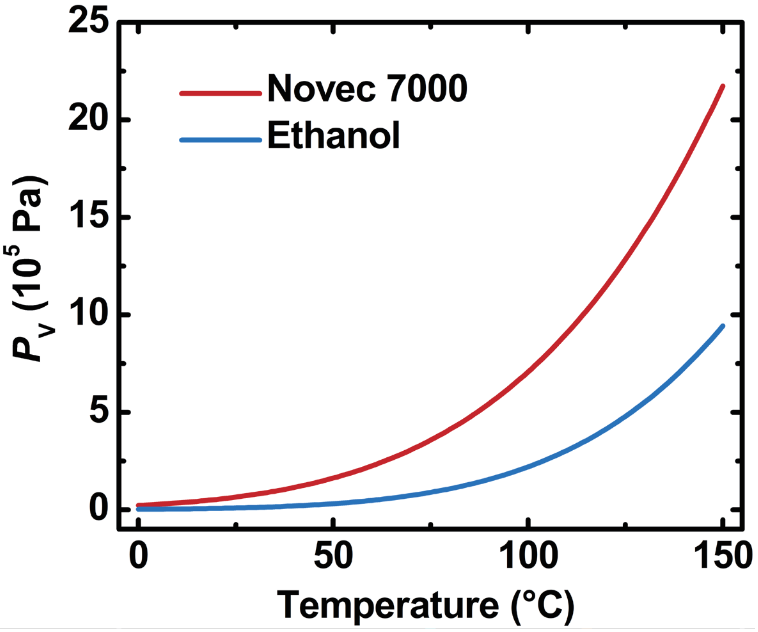

Figure S7. The saturated vapor pressure properties of ethanol and Novec 7000.

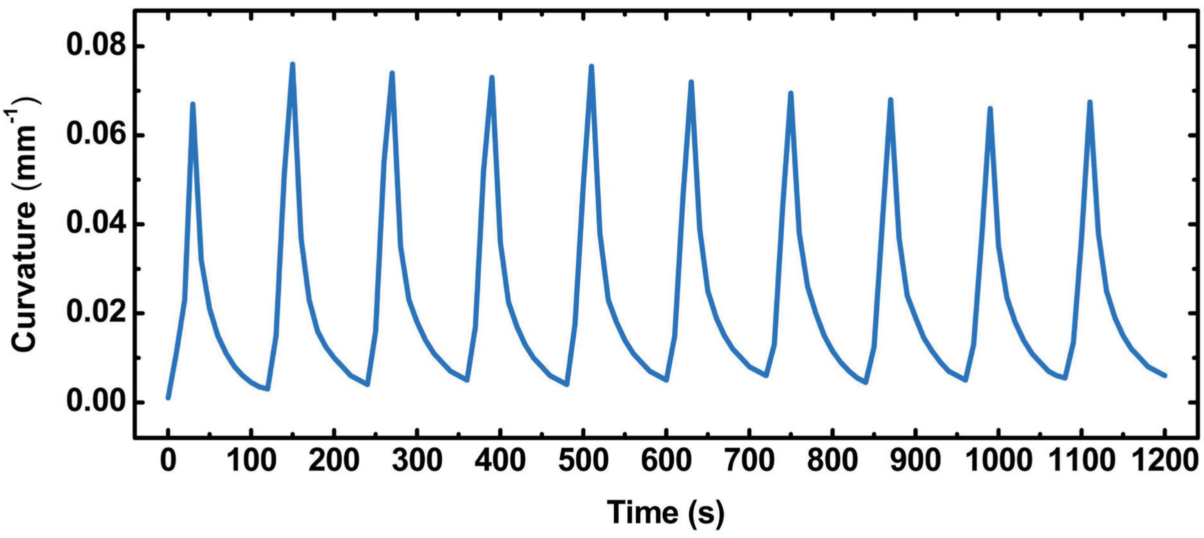

Figure S8. Cyclic bending-recovery process of the basic actuating unit of the PMMs in working mode 1. The active layer thickness was 3 mm, the strain limiting layer thickness was 1 mm and the heating power was 3.8 W. The sample had one end fixed to the fixture while operating suspended without contacting with the substrate to avoid the influence of friction.

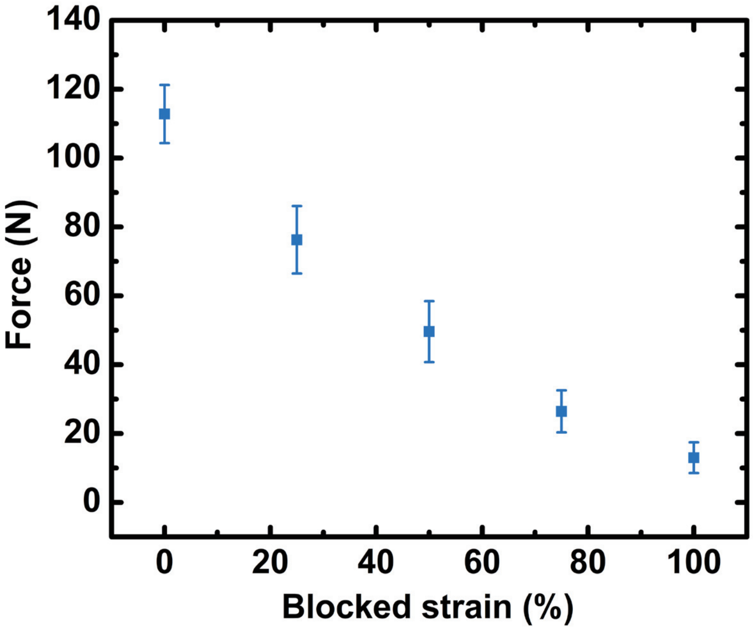

Figure S9. Blocked force at various elongations for constrained cylindrical actuation of magnetically responsive liquid-vapor phase transition composites in PMMs. The heating power was 10 W. The diameter of the cylindrical sample was 10 mm and the length was 20 mm. Five specimens were tested in each testing set.

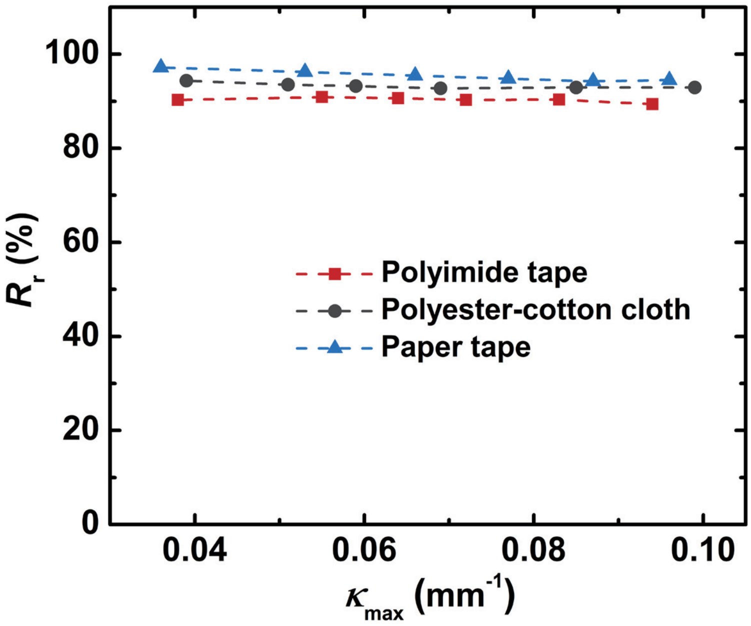

Figure S10. The effect of different substrate conditions on the deformation retention ratio *R_r* of the basic actuating unit of the PMMs. The basic actuating units operated in working mode 1. κ_{max} is the maximum curvature achieved before stopping heating. R_r is calculated using equation (5). Polyimide tape, polyester-cotton cloth or paper tape was fixed on the working board to obtain different substrate conditions. The active layer thickness was 2 mm, the strain limiting layer thickness was 1 mm.

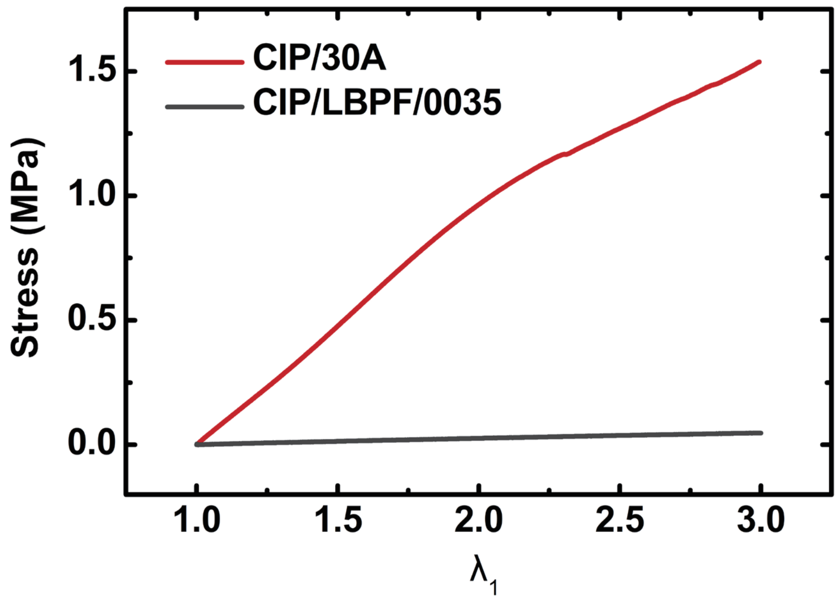

Figure S11. The stress-extension ratio relationships of the CIP/LBPF/00-35 silicone composites and the CIP/30A silicone composites in tensile tests.

Table S1. Definition of forces in the force analysis of PMMs.

Symbol	Definition of forces
$F_{\text{magnetism}}$	The magnetic force on the PMM under B
F_{support}	The support force of the substrate to the PMM
F_{friction}	The friction force of the substrate to the PMM
G	The gravity on the PMM
$F_{\text{elasticity}}$	The elastic force that tends to restore the initial shape of the basic actuating unit of the PMM under shape-locking state
$F_{\text{elasticity-}x}$	The component of $F_{\text{elasticity}}$ in the x direction
$F_{\text{elasticity-}y}$	The component of $F_{\text{elasticity}}$ in the y direction
$F_{\text{actuation}}$	The actuating force that tends to bend the basic actuating unit of the PMM under energy-storing state
$F_{\text{actuation-}x}$	The component of $F_{\text{actuation}}$ in the x direction
$F_{\text{actuation-}y}$	The component of $F_{\text{actuation}}$ in the y direction

Table S2. Comparison of PMMs, existing liquid-vapor phase transition composites, active mechanical metamaterials with shape locking, and flexible mechanical metamaterials with energy storage.

	**Active reversible deformation**	**Shape locking**	**Rapid locking and unlocking**	**Rapid actuation**	**Energy storage**	**Adjustable deformation states**	**Stretchability**	**Customizable complex structures and deformations**
Phase transition composites [1]	✓	✕	/	✕	✕	✓	✓	✕
Shape memory polymers-based active mechanical metamaterials [2]	✕	✓	✕	✕	✕	✓	✕	✓
Magnetic shape memory polymers [3]-based active mechanical metamaterials [4]	✓	✓	✕	✓	✕	✓	✕	✓
Low melting point alloys-based active mechanical metamaterials [5]	✕	✓	✕	✕	✕	✓	✕	✓
Flexible mechanical metamaterials based on bistable structures [6]	✕	✓	✓	/	✓	✕	✓	✓
**PMMs (This study)**	✓	✓	✓	✓	✓	✓	✓	✓

Note: "✓" means having this ability, while "✕" means not having this ability.

Supplementary References

1. Miriyev A, Stack K and Lipson H. Soft material for soft actuators. *Nat Commun* 2017; **8**: 596.
2. Yang C, Boorugu M and Dopp A *et al.* 4D printing reconfigurable, deployable and mechanically tunable metamaterials. *Mater Horiz* 2019; **6**: 1244-50.
3. Ze Q, Kuang X and Wu S *et al.* Magnetic shape memory polymers with integrated multifunctional shape manipulation. *Adv Mater* 2019; **32**: 1906657.
4. Ma C, Wu S and Ze Q *et al.* Magnetic multimaterial printing for multimodal shape transformation with tunable properties and shiftable mechanical behaviors. *ACS Appl Mater Interfaces* 2021; **13**: 12639-48.
5. Hwang D, Barron Edward J and Haque ABMT *et al.* Shape morphing mechanical metamaterials through reversible plasticity. *Sci Robot* 2022; **7**: eabg2171.
6. Raney JR, Nadkarni N and Daraio C *et al.* Stable propagation of mechanical signals in soft media using stored elastic energy. *Proc Natl Acad Sci USA* 2016; **113**: 9722-7.
